# Comparative study on the *in vitro* effects of *Pseudomonas aeruginosa* and seaweed alginates on human gut microbiota

**DOI:** 10.1371/journal.pone.0171576

**Published:** 2017-02-07

**Authors:** Shaofeng Bai, Huahai Chen, Liying Zhu, Wei Liu, Hongwei D. Yu, Xin Wang, Yeshi Yin

**Affiliations:** 1 State Key Laboratory of Breeding Base for Zhejiang Sustainable Pest, and Key laboratory for Food Microbial Technology of Zhejiang Province, Institute of Plant Protection and Microbiology, Zhejiang Academy of Agricultural Sciences, Hangzhou, Zhejiang, P. R. China; 2 College of Chemistry and Life Sciences, Zhejiang Normal University, Jinhua, China; 3 Departments of Biomedical Sciences, Pediatrics, Joan C. Edwards School of Medicine at Marshall University, Huntington, West Virginia, United States of America; 4 Progenesis Technologies, LLC, One John Marshall Drive, Robert C. Byrd Biotechnology Science Center, Huntington, West Virginia, United States of America; Wageningen Universiteit, NETHERLANDS

## Abstract

Alginates pertain to organic polysaccharides that have been extensively used in food- and medicine-related industries. The present study obtained alginates from an alginate overproducing *Pseudomonas aeruginosa* PAO1 mutant by screening transposon mutagenesis libraries. The interaction between bacterial and seaweed alginates and gut microbiota were further studied by using an *in vitro* batch fermentation system. Thin-layer chromatography (TLC) analysis indicated that both bacterial and seaweed alginates can be completely degraded by fecal bacteria isolated from study volunteers, indicating that a minor structural difference between bacterial and seaweed alginates (O-acetylation and lack of G-G blocks) didn’t affect the digestion of alginates by human microbiota. Although, the digestion of bacterial and seaweed alginates was attributed to different *Bacteroides xylanisolvens* strains, they harbored similar alginate lyase genes. Genus *Bacteroides* with alginate-degrading capability were enriched in growth medium containing bacterial or seaweed alginates after *in vitro* fermentation. Short-chain fatty acid (SCFA) production in both bacterial and seaweed alginates was also comparable, but was significantly higher than the same medium using starch. In summary, the present study has isolated an alginate-overproducing *P*. *aeruginosa* mutant strain. Both seaweed and bacterial alginates were degraded by human gut microbiota, and their regulatory function on gut microbiota was similar.

## Introduction

Alginates are linear, organic polysaccharides consisting of β-D-mannuronic acid (M) and α-L-guluronic acid (G). Its relative molecular weight is about 32–200 kDa [1]. Based on its properties of optimal thickening, stability, emulsification, flocculation, and chelation, alginates have been extensively used in the food, chemical, and pharmaceutical industries [2,3,4,5]. Terada *et al*. have reported that alginates increase the proliferation of fecal bifidobacterial cultures, as well as decrease specific potentially pathogenic bacterial strains (e.g., *Enterobacteriaceae* and lecithinase-negative *Clostridia*). In addition, the levels of fecal toxins produced by putrefaction (e.g., ammonia and sulfides) decreased after the administration of 10 g alginate per day in human subjects [6]. Umu *et al*. reported that replacing 5% soluble starch with alginate increases the bacterial diversity of pig gut microbiota [7]. These results thus indicate that alginates may be utilized as diet additives to influence gut microbiota and ultimately improve host health. Because of its important functions and extensive applications, there has been an increased interest in alginates around the world. In some countries, alginates are recognized as excellent food additive.

Alginates are extensively distributed in the cell walls of brown alga such as giant kelp, seaweed, silquosa, fucus and sargasso. However, alginates derived from brown algae are non-renewable. In addition, the composition of seaweed alginates varies with different breeding environments and harvest seasons [8,9]. It is thus hard to maintain the consistent quality of alginates during production. Bacterial alginates may therefore be utilized as a substitute for the development of new products and other commercial applications [10]. Two gram-negative bacterial genera, *Azotobacter* and *Pseudomonas* have been reported to have capacity of producing alginates [10]. Because the growth conditions of *Azotobacter* are stringent, its alginate production is relatively low. Moreover, the genetic manipulation tools for *Azotobacter* have not been optimized to date. Current research studies are focused on ways to regulate alginate overproduction in *Pseudomonas*. The detailed mechanisms underlying the regulation of alginate overproduction in *Pseudomonas* have been extensively studied [10,11,12,13], and genetic tools for *Pseudomonas* manipulation have been developed [13,14,15], thereby enhancing alginate yield in overproducing *Pseudomonas* strains.

In the present study, we obtained an alginate overproducing *P*. *aeruginosa* mutant by screening transposon mutagenesis libraries. We then comparatively studied the *in vitro* functions of human gut microbiota in degrading seaweed and mutant *Pseudomonas* alginates.

## Materials and methods

### Bacterial growth conditions and plasmids

*Escherichia coli* strains EC100 (Epicentre, Chicago, USA) and DH5α (TaKaRa, Dalian, China) were cultured at 37°C in Lennox broth (LB) or LB agar. *P*. *aeruginosa* strain PAO1 was grown at 37°C in LB or on *Pseudomonas* isolation agar (PIA) plates (Difco, Franklin Lakes, USA). Mariner transposon plasmid pFAC was obtained from Dr. Gao’s laboratory (Zhejiang University, Hangzhou, China). Helper plasmid pRK2013 was ordered from Lab-Bio Co. (http://www.lab-bio.com/). When required, gentamicin or kanamycin was added to the broth or plates. The concentrations of gentamicin or kanamycin added to the LB broth or plates were 20 μg mL^-1^ and 15 μg mL^-1^, respectively. The concentration of the antibiotics that were added to the PIA plates was 200 μg mL^-1^.

### Transposon mutagenesis

Conjugations were performed for transposon mutagenesis, using pFAC plasmid-carrying *E*. *coli* EC100 as the donor strain and PAO1 as the recipient strain. The transfer of plasmids from EC100 to PAO1 was performed via triparental conjugations using the helper plasmid pRK2013. After incubation, the bacteria were collected and streaked onto PIA plates supplemented with gentamicin (200 μg mL^-1^). Mucoid colonies were identified and subjected to genetic analyses. Chromosomal DNA was isolated from the mucoid mutants by using an OMEGA genomic DNA Extraction kit (OMEGA, USA). Approximately 2 μg of DNA was digested with *SalI* overnight at 37°C followed by purification and self-ligation using Fast-Link DNA ligase (Epicentre, USA). The circular closed DNA was used as template for inverse PCR using GM3OUT and GM5OUT primers [16]. The PCR products were purified and sequenced.

### Detection of the production and composition of bacterial alginate

*P*. *aeruginosa* strains were grown on triplicate PIA plates at 37°C for 24 h. The bacteria were collected and resuspended in PBS. The OD_600_ of the bacterial suspension in PBS, which corresponds to the bacterial density, was measured. The amount of uronic acid was analyzed relative to a standard curve generated using sodium alginate from brown algae (Sangon Biotech, China), as previously described [17].

Exopolysaccharides were harvested by scraping the colonies off the PIA plates and re-suspending in phosphate buffered saline (PBS). Cell pellets were removed by centrifugation, and the supernatants were combined with 3 volumes of cold ethanol (99%). Alginate fibers were collected through centrifugation and dried by speed vacuum overnight. Ten milligrams of the collected exopolysaccharide were hydrolyzed in 1 mL of 3 M trifluoroacetic acid (TFA) at 95°C for 6 h. TFA was removed from the samples by drying. The hydrolyzed sample was washed with 1 mL of methanol, and then resuspended in 10 mL of water. The composition of the alginate monomers was determined by using a Dionex ICS-5000 with a CarbopacTM PA 20 (3 × 150 mm) column and AgCl reference electrode. Water (solvent A), 500 mM NaOH (solvent B, 4%), and 250 mM NaAC (solvent C, 40%) were used as the mobile phase. A gradient of B from 4% to 40% from 15 min to 25 min was used. Standard monomers M and G, which were ordered from Qingdao BZ Oligo Biotech Co., Ltd (China), were used to establish the retention times of M and G.

### Origin of human fecal samples

A total of 5 healthy human volunteers FB, TXZ, BSF, CXX and WYS (living in Hangzhou, China), with ages ranging from 24 to 27 years old, were recruited into the study. More information related to these volunteers are listed in S1 Table. The donors had not received antibiotics and pro- or prebiotic treatment for at least 3 months prior to sample collection. All volunteers provided their informed, written consent, and the Ethics Committee of the Zhejiang Academy of Agricultural Sciences approved the study. Fresh fecal samples from these volunteers were collected immediately after defecation. A portion of each sample was stored at -80°C for DNA extraction and sequencing analysis. Another portion of each sample was homogenized in 0.1 M anaerobic phosphate-buffered saline (pH 7) to generate 10% (wt/vol) slurries for batch culture fermentation.

### *In vitro* batch culture fermentation

Batch culture fermentations were conducted using the procedure described by Rycroft *et al* [18] and Lei *et al* [19]. Briefly, the basic growth medium VI consisted of the following (g/L): yeast extract, 4.5; tryptone, 3.0; peptone, 3.0; bile salts No. 3, 0.4; L-cysteine hydrochloride, 0.8; hemin 0.05; NaCl, 4.5; KCl, 2.5; MgCl_2_.6H_2_O, 0.45; CaCl_2_.6H2O, 0.2; KH_2_PO4, 0.4; Tween 80, 1 mL; and 2 mL of a solution of trace elements. To assess the degradation and utilization of alginate by human fecal microbiota, 5.0 g of seaweed or bacterial alginate was added as the sole carbon source. The media were adjusted to pH 6.5 before autoclaving. Fresh fecal samples were homogenized in stomacher bags with 0.1 M anaerobic phosphate-buffered saline (pH 7.0) to generate 10% (wt/vol) slurries. Large food residues were removed by passing the mixture through a 0.4-mm sieve. Human fecal slurry (500 μL) was inoculated into a tube (total volume: 30 mL) containing 10 mL of growth medium and the tube was incubated at 37°C for 72 h in an anaerobic chamber (anaerobic workstation AW 500, Electrotek Ltd., UK). A 2-mL sample was collected at 24 h, 48 h, and 72 h to analyze carbohydrate degradation, bacterial community, and SCFA production.

### Thin-layer chromatography (TLC)

The degradation of alginate was detected by TLC analysis. Briefly, samples (0.2 μL) were loaded on pre-coated silica gel-60 TLC aluminum plates (Merck, Germany). After development with a solvent system consisting of formic acid/n-butanol/water (6:4:1, v:v:v), the plate was soaked in orcinol reagent and visualized by heating at 120°C for 3 min.

### Isolation and identification of alginate-degrading bacterium

A fermentation sample from volunteer BSF was collected at 72 h and then spread on an alginate agar plate (basic growth medium VI plus 5 g/L alginate and 12 g/L agar) using a 10-fold dilution method. Colonies obtained from the plates were randomly picked and re-inoculated into the alginate growth medium. TLC analysis of the supernatant was performed to assess alginate degradation. A repeat 10-fold dilution assay was performed to further purify the positive colonies.

Isolates that demonstrated an ability to degrade alginates were identified by sequencing their 16S rRNA genes. Briefly, genomic DNA was extracted by using an OMEGA Bacterial genome DNA extraction kit, and the 16S rRNA gene was PCR amplified by using primers 27F (5’-CAG AGT TTG ATC CTG GCT-3’) and 1492R (5’-AGG AGG TGA TCC AGC CGC A-3’). DNA sequencing was conducted by Shanghai Sangon Biotech Co., Ltd. (Shanghai, China). Bacterial species were identified by performing BLAST analysis of the 16S rRNA gene sequences (http://blast.ncbi.nlm.nih.gov/Blast.cgi).

### Extraction of gut microbial genomic DNA

Bacterial genomic DNA was extracted using a QIAamp DNA Stool Mini Kit following the manufacturer’s instructions (QIAGEN, Germany). The concentration of the extracted DNA was determined by using a NanoDrop ND-2000 (NanoDrop Technologies, USA), and its integrity and size were confirmed by performing 1% agar gel electrophoresis.

### PCR-DGGE analysis

To analyze the microbial communities, the V3 region of the 16S rRNA gene (positions 341 to 534 of the *E*. *coli* gene) was analyzed using PCR-denaturing gradient gel electrophoresis (DGGE), as described elsewhere [20]. DGGE was performed by using a DCode universal mutation detection system (Bio-Rad, Hercules, USA) in an 8% (wt/vol) polyacrylamide gel containing a linear 30%-to-60% denaturant gradient with a constant voltage of 200 V at 60°C for 4 h. The gels were then visualized by staining with SYBR green I nucleic acid (Sigma, USA) for 45 min and then washed twice with deionized water.

### 16S rRNA gene high-throughput sequencing and analysis

Bacterial 16S rRNA genes were amplified from the extracted DNA using barcoded primers 338F (5’- ACT CCT ACG GGA GGC AGC A-3’) with 806R (5’-GGA CTA CHV GGG TWT CTA AT-3’). Next-generation sequencing was performed on an Illumina MiSeq 300PE system that was operated by Majorbio Bio-Pharm Technology Co., Ltd., Shanghai, China.

Next-generation sequencing reads generated in the present study were identified by barcodes using a QIIME pipeline. Clean and high-quality sequences were then used for downstream analysis. A 97% similarity cutoff was employed in defining OTUs using Mothur. One sequence was picked out from each OTU as representative. The representative sequences were classified using the rdp classifier method and SILVA database. Good’s coverage, alpha diversities including Simpson and Shannon index, and richness (observed number of OTUs) were calculated using Mothur. Basic data on 16S rRNA gene high-throughput sequencing are listed in S1 Table.

### Short chain fatty acid (SCFA) analysis

SCFA production was determined by using GC as previously described [21]. Briefly, 1 mL of the fermentation products were mixed with 0.2 mL of 25% (w/v) metaphosphoric acid. The samples were subsequently centrifuged (14, 000g for 20 min), and the supernatant was used for SCFA determination (Shimadzu, GC-2010 Plus, Japan). The column of InertCap FFAP (0.25 mm×30 m×0.25 μm) was used in this study. Peaks were integrated using the GC Solution software, and SCFA content was quantified by using the single-point internal standard method. Peak identity and internal response factors were determined using a 20-mM calibration cocktail that included acetic, propionic, isobutyric, butyric, isovaleric, and valeric acids.

### Statistical analysis

To compare Illumina-based high-throughput sequencing data, Pearson correlation coefficients were analyzed using the SPSS software (version 20.0; SPSS Inc., USA). The correlation coefficients among samples were calculated based on the percentage of each bacterial classification unit at the genus level. The SCFAs of each fermentation sample were measured in triplicate. Means and standard deviations (SD) were calculated. The differences between means were assessed using ANOVA as provided by the SPSS software. P<0.05 was considered statistically significant.

### Data availability

The data generated from high-throughput 16S rRNA gene sequencing were deposited in the sequence read archive (SRA) of NCBI as GenBank Accession Number SRP082347. The bacterial 16S rRNA gene sequences of the isolated bacterial A3 and P9 were also deposited to NCBI as GenBank Accession Numbers KX658684- KX658685. The sequence of alginate lyase (*algL*) genes of A3 and P9 were submitted to NCBI as GenBank Accession Numbers KY210466-KY210467.

## Results

### Screening for *P*. *aeruginosa* mucoid mutants

**Fig 1:**
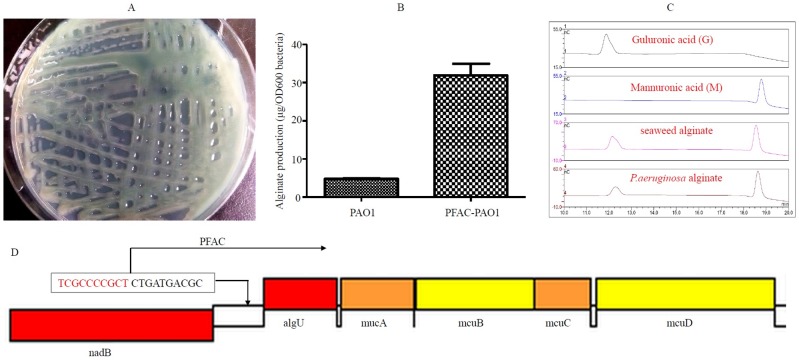
Determination of the production and composition of bacterial alginates. A mutant of *Pseudomonas aeruginosa* PAO1, which overproduces alginates, was obtained by screening transposon mutagenesis libraries. A mucoid *P*. *aeruginosa* strain (A) was used for bacterial alginate production. Alginate concentrations were measured based on the light absorption of the carbazole reaction (B). The composition of seaweed and *P*. *aeruginosa* alginates were evaluated using a DIONEX ICS5000 system (C). The monomers mannuronic acid (M) and guluronic acid (G) were used as standards. The mechanism of mucoid production was investigated by using reverse PCR and sequencing, which showed that the transposon was inserted upstream of the sigma factor gene *algU* (D), which overexpresses alginates.

To identify *P*. *aeruginosa* mucoid strains, mariner-transposon mutagenesis [22] in the non-mucoid *P*. *aeruginosa* reference strain PAO1 was performed. A mucoid variant was isolated on a PIA plate (Fig 1A), and its alginate production was determined to be substantially higher than that of PAO1 after 24 h growth at 37°C (Fig 1B). The composition of the bacterial alginate and seaweed alginate used in the present study was measured on a DIONEX ICS 5000 system. Compared to the standard alginate monomers M and G, both bacterial and seaweed alginates consisted of M and G (Fig 1C). In addition, the molecular ratio of M:G for bacterial and seaweed alginates was approximately 9:1 and 4:1, respectively. To identify the insertion site of the transposon, inverse PCR and sequencing were performed, which showed that the site of transposon insertion was located upstream of the sigma factor gene *algU*, whose upregulation induced alginate overexpression [23,24].

### Comparative utilization of seaweed and bacterial alginates by 5 human fecal samples

**Fig 2:**
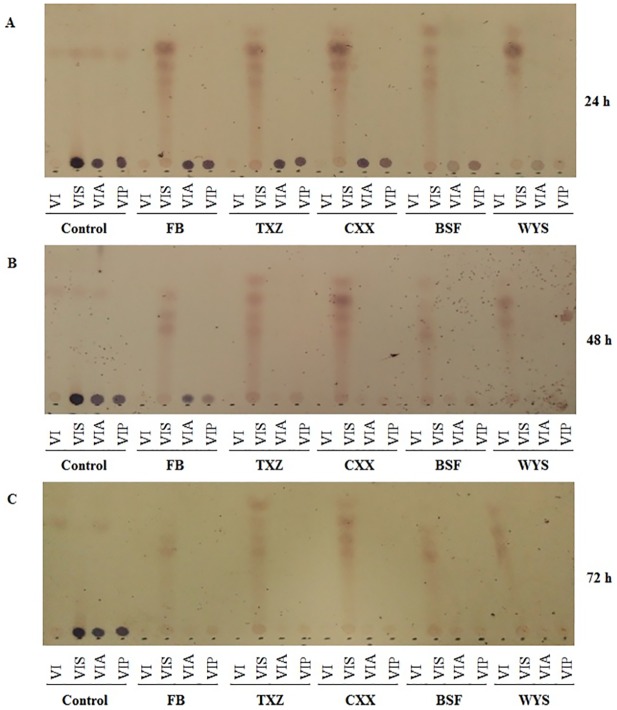
The degradation of seaweed and bacterial alginates by human gut microbiota. The degradation of alginates by human gut microbiota was evaluated at different time points in the batch chemostat system using TLC. The control group indicates no microbiota was inoculated; FB, TXZ, CXX, BSF, and WYS represent fecal bacteria from these volunteers that were inoculated in the batch chemostat; the letters VI, VIS, VIA, and VIP on the TLC plate represent VI media, VI media plus soluble starch, VI media plus seaweed alginate, and VI media plus bacterial alginate, respectively.

*P*. *aeruginosa* alginates were prepared by alcohol precipitation and vacuum drying. Seaweed alginate was ordered from Sangon Biotech (China). Alginate degradation varied with initial inocula and fermentation times. The gut microbiota obtained from volunteers BSF and WYS degraded both bacterial and seaweed alginates after 24 h of fermentation. The gut microbiota of volunteers TXZ and CXX degraded both bacterial and seaweed alginates after 48 h of fermentation. For sample FB, degradation was detected after 72 h of fermentation (Fig 2). For the control, the dot color of the no-carbon source added group was very thin on the TLC plates, and soluble starch was also degraded by human gut microbiota.

### Isolation of alginate-degrading bacteria

**Fig 3:**
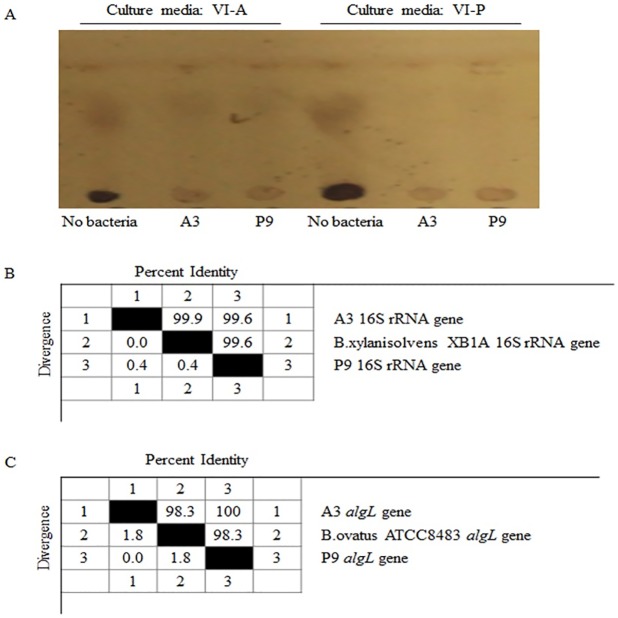
Isolation and identification of alginate-degrading bacteria. The fermentation products of sample BSF were used in the isolation of alginate-degrading bacteria. Seaweed alginate-degrading bacteria A3 was isolated from the plates of VI plus seaweed alginate. Bacterial alginate-degrading bacteria P9 was isolated from the plates of VI plus *P*. *aeruginosa* alginate. The degradation function of A3 and P9 on alginates was detected by using TLC (A). VIA, culture media of VI plus seaweed alginate; VIP, culture media of VI plus *P*.*aeruginosa* alginate; No bacteria were represent in the control; A3 and P9, alginate-degrading bacterial strains A3 and P9 were inoculated. The 16S rRNA genes and alginate lyases genes of the bacterial strains A3 and P9 were identified by using PCR and sequencing. The similarity of the 16S rRNA gene (B) and alginate lyase gene (C) between A3 to P9 was analyzed by using the DNAStar software.

Because the gut microbiota isolated from volunteer BSF showed the highest rate of alginate degradation, the fermentation products, which had been grown in the medium containing alginate for 72 h, were spread on alginate agar plates for the isolation of alginate-hydrolyzing bacteria. Colonies were randomly selected from the alginate agar plates and re-inoculated into the VI growth medium containing 5 g of alginate. TLC analysis was conducted after 72 h of fermentation. TLC results indicated that the seaweed and bacterial alginate substrates were completely depleted from the supernatant of isolates A3 and P9. The positive colony was then purified by repeated spreading on the alginate agar plate in the anaerobic chamber and then stored at -80°C until further use. The degradation by these bacteria on different sourced alginate was also assessed. Fig 3A shows that both A3 and P9 degraded bacterial and seaweed alginates.

To taxonomically classify the alginate-degrading bacteria A3 and P9, 16S rRNA gene universal primers 27F and 1492R were used for PCR amplification and sequencing. Both A3 and P9 were identified as *Bacteroides xylanisolvens* and BLAST analysis showed that it was 99% similar to that of the *B*. *xylanisolvens* XB1A strain. The 16S rRNA genes of A3 and P9 showed 99.6% identity (Fig 3B). Bioinformatics analysis identified an alginate lyase gene in *B*. *ovatus* strain ATCC 8483. A pair of primers for PCR amplification of this gene was designed (*algL*-F: 5’- ttt ttg ccc tga ctt cta cca-3’; *algL*-R: 5’-ggt cat aac cac cgc tat tag-3’). The genes encoded in A3 and P9 were analyzed by PCR and clone library sequencing. Sequence alignment showed that the alginate lyase genes of A3 and P9 were 100% homologous (Fig 3C).

### Effects of alginate on the composition of human fecal microbiota communities

**Fig 4:**
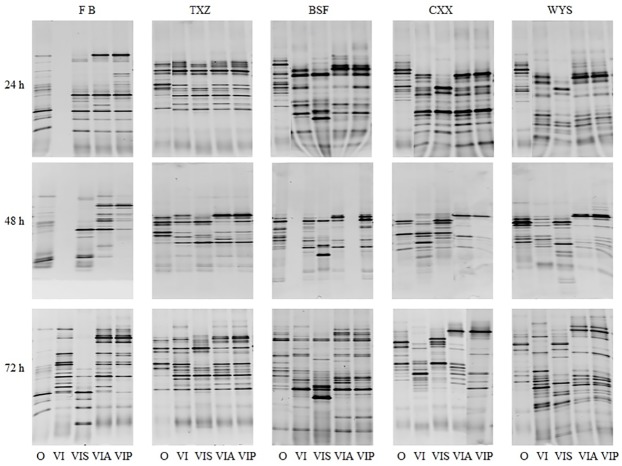
PCR-DGGE analysis of the effects of alginates on human gut microbiota. Batch fermentation products were collected at 24 h, 48 h and 72 h after inoculation. Bacterial genomic DNA was extracted and used in PCR-DGGE analysis. FB, TXZ, BSF, CXX and WYS represent fecal bacterial samples that were inoculated in the batch chemostat. O, the original fecal samples; VI, VIS, VIA, and VIP, samples collected after cultured using VI media, VI media plus soluble starch, VI media plus seaweed alginates and VI media plus bacterial alginates, respectively.

To compare the effects of alginates on the composition of fecal microbiota, changes in the bacteria communities of 5 fecal samples were measured using PCR-DGGE after 24 h, 48 h and 72 h of fermentation. Fig 4 shows that the number of DGGE bands in most of the samples increased with fermentation time. In general, the number of bands observed at 72 h was higher than that at 24 h and 48 h of fermentation. For the starch-added group, the bacterial communities in the fermentation products were clearly distinct from the other groups after culturing for 72 h. However, similar patterns between seaweed and bacterial alginate-added groups were detected for the bacterial communities detected in all volunteers (Fig 4).

**Fig 5:**
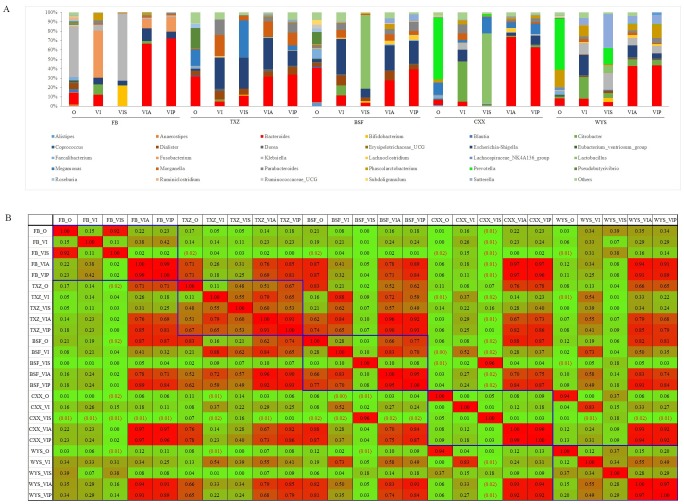
The effect of alginates on human gut microbiota by high-throughput 16S rRNA gene sequencing. Batch fermentation products, which were collected at 72 h after inoculation, were sent for 16S rRNA gene high-throughput sequencing. The microbial communities at the genus level were analyzed (A). O, original fecal samples; VI, VIS, VIA, and VIP, samples collected after cultured using VI media, VI media plus soluble starch, VI media plus seaweed alginates and VI media plus bacterial alginates, respectively. To compare pyrosequencing data, Pearson correlation coefficients were analyzed by using the SPSS 20.0 software (B).

The fermentation products collected at 72 h were also used in 16S rRNA gene V3-V4 region high-throughput sequencing. Fig 5A shows that the bacterial communities between the seaweed and alginate groups were highly similar and mainly comprised of *Bacteroides*. However, the bacterial community of the original inocula differed. The predominant genus in the FB’s fecal sample was *Klebsiella*, whereas that in fecal samples BSF and TXZ was *Bacteroides*, and that in CXX and WYS was *Prevotella*. *Bacteroides* were enriched in all of the fermentation products that were mixed with seaweed or bacterial alginates. However, the density of *Bacteroides* in the no-sugar or soluble starch added group was relatively very low. The similarity coefficient between the original fecal samples and the fermentation products varied with the original bacterial community and culture media (Fig 5B). The addition of starch enhanced the proliferation of fecal microbiota in sample FB, and the similarity coefficient was as high as 0.92. The capacity to stimulate the proliferating of fecal microbiota in samples BSF and TXZ in the bacterial alginate-added group was apparently superior than that of the other groups. The similarity coefficient between the original microbiota and the fermentation products was 0.67 and 0.77 for fecal samples TXZ and BSF, respectively. In addition, all culture media did not stimulate the proliferation of fecal microbiota from samples CXX and WYS. For VI group that had no carbon source added, the bacterial community of the fermentation products showed distinct differences from their original bacterial communities.

### Detection of SCFA production

**Fig 6:**
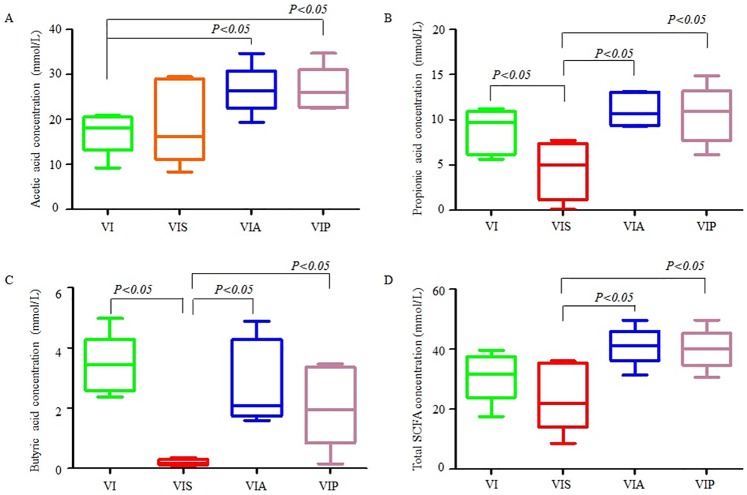
The effects of alginate on SCFA production after 72 h of fermentation. The effects of different culture media on SCFA production in the batch chemostat was assessed using gas chromatography (GC). Acetic, propionic, isobutyric, butyric, isovaleric, and valeric acids were detected in the present study, and the total SCFA represents all SCFAs. VI, VIS, VIA and VIP represent samples collected after cultured using VI media, VI media plus soluble starch, VI media plus seaweed alginates and VI media plus bacterial alginates, respectively. The SCFAs of each fermentation sample were measured in triplicate. Box plot figures were generated using GraphPad Prism Version 5.01. The differences between means were assessed using ANOVA as provided in the SPSS 20.0 software.

Before inoculation, the SCFA concentrations of the original fecal samples were measured. The average concentrations of acetic, propionic, butyric, isobutyric, valeric acids, and isovaleric acids in the fermentation tubes were 0.052, 0.022, 0.001, 0.016, 0.002, and 0.001 mmol/L, respectively. SCFA concentrations increased after fermentation, and SCFA production increased with fermentation time (S1 Fig). After 24 and 48 h of fermentation, the total SCFAs in each group did not show significant differences (S2 and S3 Figs). At 72 h of fermentation, the propionic, butyric and total SCFA concentrations were higher in the alginates-added groups than that of the soluble starch-added group. However, SCFA production of acetic, propionic, butyric, and total SCFA was similar between the seaweed and bacterial alginate groups (Fig 6).

## Discussion

Seaweed alginates have been extensively employed in the manufacturing industry as food additives and modified release capsules [2,3,4]. However, alginate production from seaweed is non-renewable and structurally variable [7,8]. Alginates generated from bacteria maybe an alternate method based on their compositional homogeneity and reproducibility [13]. In the present study, we isolated an alginate-overproducing *P*. *aeruginosa* mutant strain by screening mutagenesis libraries. Compared to seaweed alginates, bacterial alginates were also degraded by the microbiota from all volunteers. In addition, the effect of bacterial and seaweed alginates on gut microbiota was similar. However, the G content of *Pseudomonas* alginates is lower than that of seaweed alginates [1], which is consistent with our HPLC data (Fig 1). The biochemical features of these two alginates thus requires further investigation. To the best of our knowledge, no G-G blocks have been reported in *Pseudomonas* alginates. To potentially substitute brown alginates, it is necessary to increase G block formation in *Pseudomonas* alginates. Previous studies have shown that *Azotobacter* epimerase genes are involved in the G block formation [25,26]. In theory, increasing the G content and G block of *Pseudomonas* alginates is feasible in the field of synthetic microbiology.

In the present study, TLC analysis indicated that both seaweed and bacterial alginates can be degraded by fecal bacteria. Although, the bacteria isolated from volunteer BSF for the degradation of bacterial and seaweed alginates may be different *B*. *xylanisolvens* strains, they harbor the same lysis genes. We then analyzed the presence of *B*. *xylanisolvens* sequences in our microbiota data using BLAST and the presence of the *algL* gene using PCR. As a result, the sequence of OTU15 in the representation OTUs is belong to *B*. *xylanisolvens*. Although, *B*. *xylanisolvens* was detected in all samples, the *algL* genes were not identified in some samples, particularly in the starch-added group (S2 Table). This discrepancy may be due to the fact that not all *B*. *xylanisolvens* strains harbor the *algL* gene. Furthermore, BLAST analysis using the *algL* gene sequence in NCBI website, no *B*. *xylanisolvens* was found to encode this similar gene in their genome sequences. The strain specifically also has been reported exist widely in gut microbiota in previous studies [27]. Despite the fact that all fermented bacteria in this study can degrade both seaweed and bacterial alginate, the *algL* gene was not detected in the original FB and TXZ fecal samples. Two reasons may explain the observed phenomenon. One is that the relative quantity of the *algL* gene is very low in these two samples that therefore generates false-negative PCR results. TLC analysis also showed that the degradation rate of the FB group was lower (Fig 2). The second is that the degradation of alginate in volunteers FB and TXZ may be caused by additional alginate lysis genes. Several different bacterial species such as *Formosa haliotis*, *Pseudoalteromonas sp*., and *Flammeovirga sp*. have been reported to degrade alginates [28,29,30]. The sequence homology of the lysis genes from different bacterial species is relatively low; for example, *P*. *protegens* Pf-5 and *P*. *aeruginosa* PAO1 show only a 74.4% similarity in the *algL* genes.

Alginates are natural macromolecules composed of β-D-mannuronate and α-L-guluronate units linked by 1→4 glycosidic bonds. In addition, carbohydrates are important nutritive materials. Several polysaccharides, such as inulin, lactulose, fructose, and resistant starch have been utilized as prebiotics [31,32]. Alginates are also potential prebiotics. Terada *et al*. have reported that alginates can regulate the growth and proliferation of colonic bacteria [6]. In the present study, we found that the microbial community in alginate-added groups was distinctly different from the starch-added group. Although previous studies have shown that different enterotypes exist among individuals [29,30], the present study showed that alginate regulation did not depend on the original fecal bacterial community as *Bacteroides* was enriched in all fermentations. Moreover, SCFA production in the bacterial and seaweed alginates-added groups was significantly higher than that of the starch-added group. So far, most researchers have reported that increasing the concentration of gut SCFA improves host health [33,34]. Therefore, further investigations of the prebiotic effects of alginate are warranted.

In summary, bacterial alginates were produced by a *P*. *aeruginosa* mutant strain. Both seaweed and bacterial alginates were degraded by the human gut microbiota. The effect of bacterial and seaweed alginates on gut microbiota was similar. The findings of the present study may serve as a foundation for potential replacement of seaweed alginates with bacterial alginates in terms of prebiotics development.

## Supporting information

S1 Fig The effects of fermentation times on bacterial SCFA production. The effects of fermentation times (24 h, 48 h, and 72 h) on SCFA production (acetic, propionic, isobutyric, butyric, isovaleric, and valeric acids) in a batch chemostat were assessed by using gas chromatograph (GC). Total SCFA represents all SCFAs. VI, VIS, VIA and VIP indicate the samples collected after culturing in VI media, VI media plus soluble starch, VI media plus seaweed alginates and VI media plus bacterial alginates, respectively. The SCFAs in each fermentation sample were measured in triplicate, and the means were calculated.
(TIF)

S2 Fig The effects of different culture media on SCFA production after 24 h fermentation. The fermentation products at 24 h were collected for GC detection. Acetic, propionic, isobutyric, butyric, isovaleric, and valeric acid were detected, and total SCFA represents all SCFAs. VI, VIS, VIA and VIP represent the samples collected after culturing in VI media, VI media plus soluble starch, VI media plus seaweed alginates and VI media plus bacterial alginates, respectively. The SCFAs in each fermentation sample were measured in triplicate. Box plot figures were generated using GraphPad Prism Version 5.01. Differences among means were assessed using ANOVA as provided in the SPSS 20.0 software.
(TIF)

S3 Fig The effects of different culture media on SCFA production after 48 h fermentation. The fermentation products at 48 h were collected for GC detection. Acetic, propionic, isobutyric, butyric, isovaleric, and valeric acid were detected, and total SCFA represents all SCFAs. VI, VIS, VIA and VIP represent the samples collected after culturing in VI media, VI media plus soluble starch, VI media plus seaweed alginates and VI media plus bacterial alginates, respectively. The SCFAs in each fermentation sample were measured in triplicate. Box plot figures were generated using GraphPad Prism Version 5.01. Differences among means were assessed by using ANOVA as provided in the SPSS 20.0 software.
(TIF)

S1 Table Basic information on the volunteers. (DOC)

S2 Table Distribution of B. xylanisolvens and its algL gene in the original fecal and fermented samples. (DOC)
