# Supplementary figures and images for: Comparative study on the in vitro effects of Pseudomonas aeruginosa and seaweed alginates on human gut microbiota
Source: PLoS One. 2017 Feb 7;12(2):e0171576. doi: 10.1371/journal.pone.0171576 (PMC5295698; doi:10.1371/journal.pone.0171576)

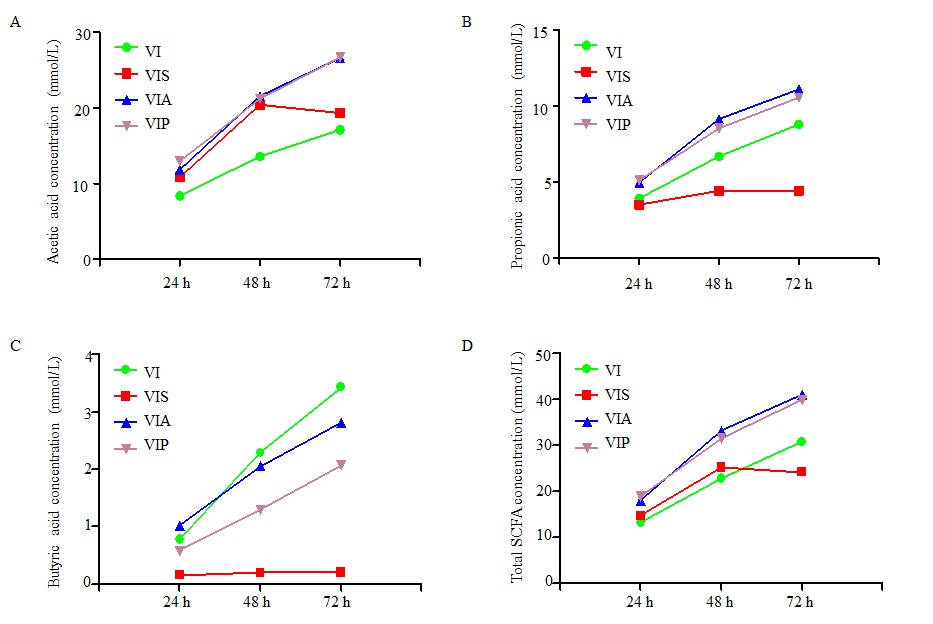

Supplement: S1 Fig — The effects of fermentation times (24 h, 48 h, and 72 h) on SCFA production (acetic, propionic, isobutyric, butyric, isovaleric, and valeric acids) in a batch chemostat were assessed by using gas chromatograph (GC). Total SCFA represents all SCFAs. VI, VIS, VIA and VIP indicate the samples collected after culturing in VI media, VI media plus soluble starch, VI media plus seaweed alginates and VI media plus bacterial alginates, respectively. The SCFAs in each fermentation sample were measured in triplicate, and the means were calculated. (TIF) [file pone.0171576.s001.tif]

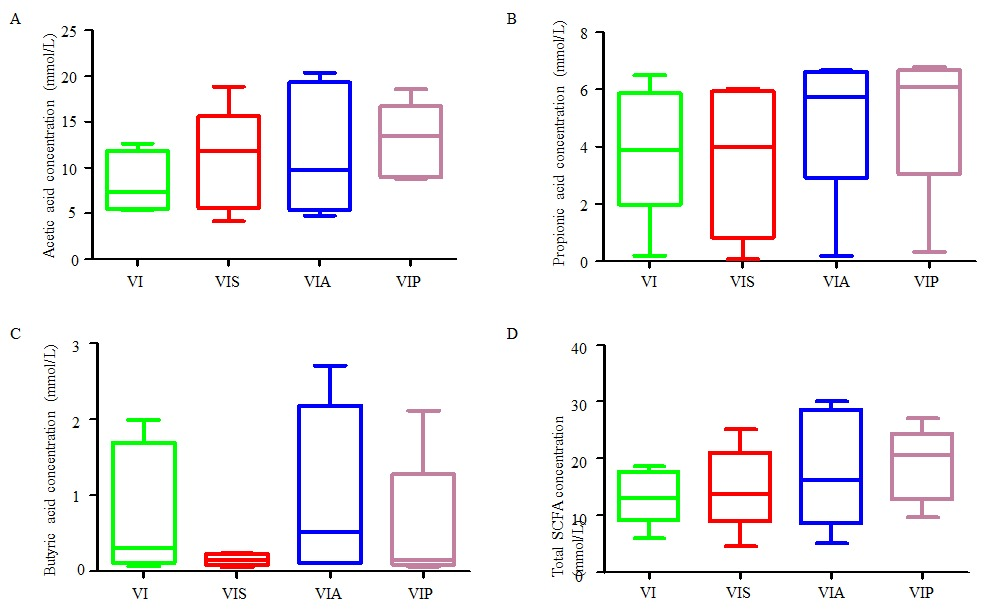

Supplement: S2 Fig — The fermentation products at 24 h were collected for GC detection. Acetic, propionic, isobutyric, butyric, isovaleric, and valeric acid were detected, and total SCFA represents all SCFAs. VI, VIS, VIA and VIP represent the samples collected after culturing in VI media, VI media plus soluble starch, VI media plus seaweed alginates and VI media plus bacterial alginates, respectively. The SCFAs in each fermentation sample were measured in triplicate. Box plot figures were generated using GraphPad Prism Version 5.01. Differences among means were assessed using ANOVA as provided in the SPSS 20.0 software. (TIF) [file pone.0171576.s002.tif]

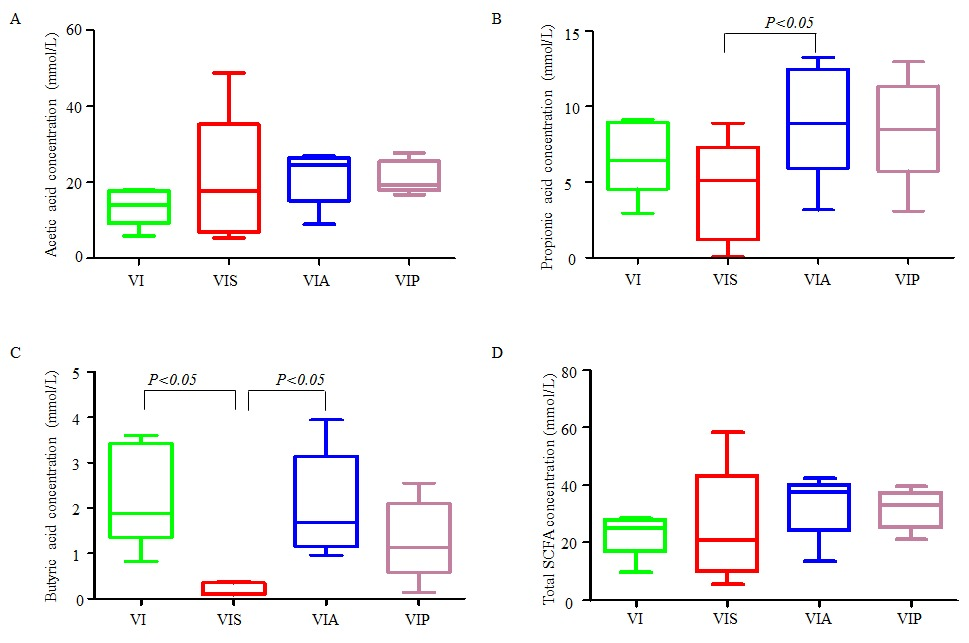

Supplement: S3 Fig — The fermentation products at 48 h were collected for GC detection. Acetic, propionic, isobutyric, butyric, isovaleric, and valeric acid were detected, and total SCFA represents all SCFAs. VI, VIS, VIA and VIP represent the samples collected after culturing in VI media, VI media plus soluble starch, VI media plus seaweed alginates and VI media plus bacterial alginates, respectively. The SCFAs in each fermentation sample were measured in triplicate. Box plot figures were generated using GraphPad Prism Version 5.01. Differences among means were assessed by using ANOVA as provided in the SPSS 20.0 software. (TIF) [file pone.0171576.s003.tif]
