# Supplementary material for: Comparative study on the in vitro effects of Pseudomonas aeruginosa and seaweed alginates on human gut microbiota
Source: PLoS One. 2017 Feb 7;12(2):e0171576. doi: 10.1371/journal.pone.0171576 (PMC5295698; doi:10.1371/journal.pone.0171576)
Supplement: S1 Table — (DOC) [file pone.0171576.s004.doc]

**S1 Table. Basic information on the volunteers.**

| Volunteer | Sample ID | Age (years) | Sex | Reads | OTU (0.97) | Ace | Chao | Coverage | Shannon | Simpson |
| --- | --- | --- | --- | --- | --- | --- | --- | --- | --- | --- |
| BSF | BSF20151020 | 23 | Male | 38458 | 232 | 275 | 288 | 0.998856 | 3.67 | 0.0467 |
| CXX | CXX20151020 | 25 | Female | 47103 | 205 | 228 | 236 | 0.999278 | 2.3 | 0.2067 |
| FB | FB20151020 | 26 | Male | 51570 | 176 | 205 | 205 | 0.999399 | 2.39 | 0.2324 |
| TXZ | TXZ20151020 | 25 | Male | 44441 | 204 | 249 | 269 | 0.998965 | 2.69 | 0.1207 |
| WYS | WYS20151020 | 25 | Female | 48713 | 197 | 217 | 223 | 0.999384 | 2.6 | 0.1464 |
