# Supplementary material for: Comparative study on the in vitro effects of Pseudomonas aeruginosa and seaweed alginates on human gut microbiota
Source: PLoS One. 2017 Feb 7;12(2):e0171576. doi: 10.1371/journal.pone.0171576 (PMC5295698; doi:10.1371/journal.pone.0171576)
Supplement: S2 Table — (DOC) [file pone.0171576.s005.doc]

**S2 Table. Distribution of *B. xylanisolvens* and its *algL* gene in the original fecal and fermented samples.**

| Sample ID | *B. xylanisolvens* %^a^ | *algL* PCR^b^ |
| --- | --- | --- |
| FB_O | 0.10% | N |
| TXZ_O | 0.21% | N |
| CXX_O | 0.12% | P |
| BSF_O | 1.13% | P |
| WYS_O | 0.22% | P |
| FB_VI | 1.12% | P |
| FB_VIS | 0.01% | N |
| FB_VIA | 33.75% | P |
| FB_VIP | 36.51% | N |
| TXZ_VI | 0.32% | P |
| TXZ_VIS | 0.03% | N |
| TXZ_VIA | 0.25% | P |
| TXZ_VIP | 0.82% | P |
| CXX_VI | 0.05% | P |
| CXX_VIS | 0.00% | N |
| CXX_VIA | 0.13% | P |
| CXX_VIP | 0.15% | P |
| BSF_VI | 0.10% | P |
| BSF_VIS | 0.02% | N |
| BSF_VIA | 4.56% | P |
| BSF_VIP | 1.43% | P |
| WYS_VI | 0.09% | P |
| WYS_VIS | 0.85% | P |
| WYS_VIA | 15.90% | P |
| WYS_VIP | 14.40% | P |

^a^ V3-V4 region 16S rRNA gene of the isolated A3 and P9 was used for BLAST analysis using OTU representative sequences.

^b^ Primers targeting the Bacteroides *algL* gene were used in PCR detection.
